# The Muscle Protein Synthetic Response to the Ingestion of a Plant-Derived Protein Blend Does Not Differ from an Equivalent Amount of Milk Protein in Healthy Young Males

**DOI:** 10.1093/jn/nxac222

**Published:** 2022-09-28

**Authors:** Philippe J M Pinckaers, Imre W K Kouw, Stefan H M Gorissen, Lisanne H P Houben, Joan M Senden, Will K H W Wodzig, Lisette C P G M de Groot, Lex B Verdijk, Tim Snijders, Luc J C van Loon

**Affiliations:** TiFN, Wageningen, The Netherlands; NUTRIM School of Nutrition and Translational Research in Metabolism, Department of Human Biology, Maastricht University Medical Centre+, Maastricht, The Netherlands; TiFN, Wageningen, The Netherlands; NUTRIM School of Nutrition and Translational Research in Metabolism, Department of Human Biology, Maastricht University Medical Centre+, Maastricht, The Netherlands; NUTRIM School of Nutrition and Translational Research in Metabolism, Department of Human Biology, Maastricht University Medical Centre+, Maastricht, The Netherlands; NUTRIM School of Nutrition and Translational Research in Metabolism, Department of Human Biology, Maastricht University Medical Centre+, Maastricht, The Netherlands; TiFN, Wageningen, The Netherlands; NUTRIM School of Nutrition and Translational Research in Metabolism, Department of Human Biology, Maastricht University Medical Centre+, Maastricht, The Netherlands; Central Diagnostic Laboratory, Maastricht University Medical Centre+, Maastricht, The Netherlands; TiFN, Wageningen, The Netherlands; Division of Human Nutrition & Health, Department of Agrotechnology and Food Sciences, Wageningen University, Wageningen, The Netherlands; TiFN, Wageningen, The Netherlands; NUTRIM School of Nutrition and Translational Research in Metabolism, Department of Human Biology, Maastricht University Medical Centre+, Maastricht, The Netherlands; TiFN, Wageningen, The Netherlands; NUTRIM School of Nutrition and Translational Research in Metabolism, Department of Human Biology, Maastricht University Medical Centre+, Maastricht, The Netherlands; TiFN, Wageningen, The Netherlands; NUTRIM School of Nutrition and Translational Research in Metabolism, Department of Human Biology, Maastricht University Medical Centre+, Maastricht, The Netherlands

**Keywords:** muscle protein synthesis, plant-based proteins, dairy, protein blends, fractional synthesis rate, young healthy males

## Abstract

**Background:**

Plant-derived proteins are considered to have lesser anabolic properties when compared with animal-derived proteins. The attenuated rise in muscle protein synthesis rates following ingestion of plant-derived compared with animal-derived protein has been, at least partly, attributed to deficiencies in specific amino acids such as leucine, lysine, and/or methionine. Combining different plant-derived proteins could provide plant-derived protein blends with a more balanced amino acid profile.

**Objectives:**

This study aimed to compare postprandial muscle protein synthesis rates following the ingestion of 30 g milk protein with a 30 g blend combining wheat, corn, and pea protein in healthy young men.

**Methods:**

In a randomized, double-blind, parallel-group design, 24 young males (aged 24 ± 4 y) received a primed continuous l-[ring-^13^C_6_]-phenylalanine infusion after which they ingested 30 g milk protein (MILK) or a 30 g plant-derived protein blend combining 15 g wheat, 7.5 g corn, and 7.5 g pea protein (PLANT-BLEND). Blood and muscle biopsies were collected frequently for 5 h to assess postprandial plasma amino acid profiles (secondary outcome) and subsequent muscle protein synthesis rates (primary outcome). Data were analyzed by 2-factor repeated measures ANOVA and 2-samples *t* tests.

**Results:**

MILK increased plasma essential amino acid concentrations more than PLANT-BLEND over the 5 h postprandial period (incremental AUC = 151 ± 31 compared with 79 ± 12 mmol·300 min·L^−1^, respectively; *P* < 0.001). Ingestion of both MILK and PLANT-BLEND increased myofibrillar protein synthesis rates (*P* < 0.001), with no significant differences between treatments (0.053 ± 0.013%/h and 0.064 ± 0.016%/h, respectively; *P* = 0.08).

**Conclusions:**

Ingestion of 30 g plant-derived protein blend combining wheat-, corn-, and pea-derived protein increases muscle protein synthesis rates in healthy young males. The muscle protein synthetic response to the ingestion of 30 g of this plant-derived protein blend does not differ from the ingestion of an equivalent amount of a high-quality animal-derived protein.

Clinical trial registry number for Nederlands Trial Register: NTR6548 (https://trialsearch.who.int/Trial2.aspx?TrialID=NTR6548).

## Introduction

Protein ingestion increases muscle protein synthesis rates (1, 2). The increase in muscle protein synthesis rate is driven by the postprandial increase in circulating plasma essential amino acid (EAA) concentrations (3), with the rise in circulating leucine being of particular relevance (4–8). The anabolic properties of different proteins or protein sources seem to be largely determined by their protein digestion and amino acid absorption kinetics, EAA content, and amino acid profile (9–11). Consequently, postprandial muscle protein synthesis rates can differ substantially following ingestion of the same amount of protein derived from different sources (12–14).

Plant-based proteins comprise a large part of our daily protein intake (15) and are likely to become more important with the global transition toward consumption of a more plant-based protein diet (16, 17). However, plant-derived proteins are believed to have lesser anabolic properties when compared with animal-derived proteins due to their lower digestibility and/or incomplete amino acid profile (17, 18). Most plant-derived proteins are relatively low in EAA content and often show deficiencies in ≥1 specific amino acids, such as leucine, lysine, and/or methionine (19). Combining different plant-derived proteins within a single blend represents one of the strategies to compose a bolus of plant-derived proteins with a more balanced amino acid profile, with less apparent amino acid deficiencies (17–22). Whereas some plant-based proteins are particularly deficient in lysine, others are deficient in methionine (19). Combining corn-, hemp-, or brown rice–derived protein (low lysine and high methionine content) with soy- or pea-derived protein (low methionine and high lysine content) provides us with the opportunity to compose blends of proteins that complement each other for their amino acid deficiencies (18–22). As such, plant-based protein blends can provide amino acid profiles that closely resemble high-quality animal-derived proteins, with fewer amino acid deficiencies compared with individual plant-based proteins.

Previous work has shown that blends of animal- and plant-derived proteins can be as effective as high-quality animal-derived proteins to increase muscle protein synthesis during recovery from exercise (21, 23). To our knowledge, no studies have compared the anabolic properties of an exclusively plant-derived protein blend with a high-quality animal-derived protein when ingested in a resting condition. Therefore, we composed a plant-derived protein blend with an amino acid composition that is similar to most animal-derived proteins, combining a high leucine content and no apparent amino acid deficiencies. By combining wheat and corn protein [with lysine contents below WHO/FAO/United Nations University (UNU) requirements (24)] with pea protein (with lysine content being amongst the highest for plant-derived proteins) we composed a protein blend with no apparent lysine deficiency. Furthermore, whereas wheat- and pea-derived proteins fall short for the WHO/FAO/UNU methionine requirements (25), corn protein can compensate for this with its high methionine content. Finally, the leucine content of corn exceeds even the concentrations observed in whey protein and, as such, can be used to create a plant-derived protein blend with a high leucine content (19).

We hypothesized that the ingestion of a plant-based protein blend consisting of wheat-, corn-, and pea-derived protein, could strongly increase muscle protein synthesis rates. Furthermore, we hypothesized that the muscle protein synthetic response to the ingestion of this protein blend would not be inferior when compared with a high-quality animal-derived protein such as milk protein. To test our hypotheses, we selected 24 healthy young men to take part in this study, in which we compared the impact of ingesting 30 g milk protein with the ingestion of an equivalent amount of a plant-based protein blend (providing 15 g wheat protein, 7.5 g corn protein, 7.5 g pea protein) on in vivo postprandial muscle protein synthesis rates.

## Methods

### Participants

Healthy, recreationally active males aged 18–35 y inclusive were eligible to participate in this parallel-group, double-blind, randomized controlled trial (participants’ characteristics are presented in **Table 1**). Participants were recreationally active and generally performed 2–4 exercise sessions per week in various sports (soccer, basketball, weight lifting, running, cycling, etc.), but were not involved in any structured progressive exercise training regimen. This study was part of a larger trial registered at the Netherlands Trial Register (NTR6548), and was conducted between June 2017 and April 2019 at Maastricht University in Maastricht, The Netherlands (see **Supplemental Figure 1** for the CONSORT flow diagram, indicating the specific comparison that the current study was based on). The data of the milk protein group have been published previously, as well as the procedures applied in this trial (26). All participants were informed about the purpose of the study, the experimental procedures, and possible risks before providing written informed consent to participate. The procedures followed were in accordance with the ethical standards of the Medical Ethics Committee of Maastricht University Medical Centre+ (METC 173001), and in accordance with the Helsinki Declaration of 1975 as revised in October 2013. The study was independently monitored and audited by the Clinical Trial Centre Maastricht.

**TABLE 1:** Participants’ characteristics^1^

	MILK	PLANT-BLEND
Age, y	26 ± 4	22 ± 4
Height, m	1.76 ± 0.06	1.80 ± 0.06
Weight, kg	71.5 ± 9.0	70.8 ± 7.9
BMI, kg/m^2^	23.0 ± 2.1	21.9 ± 2.2
Systolic blood pressure, mmHg	119 ± 6	129 ± 7
Diastolic blood pressure, mmHg	71 ± 9	68 ± 8
Resting heart rate, bpm	64 ± 10	64 ± 8
Lean body mass, kg	53.2 ± 7.9	54.0 ± 5.3
Body fat, %	23.1 ± 3.2	20.9 ± 4.4

1 Values represent mean ± SD; *n* = 12 per nutritional intervention group. MILK: 30 g milk protein; PLANT-BLEND: 15 g wheat protein + 7.5 g corn protein + 7.5 g pea protein. Two-samples *t* test all *P* > 0.05.

### Preliminary testing

Participants aged 18–35 y, with BMI >18.5 and <27.5 kg/m^2^, underwent an initial screening session to assess eligibility. Height, weight, blood pressure, and body composition (by DXA; Discovery A, Hologic; NHANES—body composition analysis enabled) were determined. Participants were deemed healthy based on their responses to a medical questionnaire. The screening sessions and experimental trials were separated by ≥3 d.

### Study design

Participants were randomly assigned to ingest a 400 mL beverage containing either 30 g milk protein concentrate (MILK) or a 30 g plant-protein blend consisting of 15 g wheat protein hydrolysate, 7.5 g corn protein isolate, and 7.5 g pea protein concentrate (PLANT-BLEND). After beverage ingestion, the bottle was rinsed with 150 mL water, which was also ingested by the participants. Milk protein concentrate (Refit MPC80) was obtained from FrieslandCampina, wheat protein hydrolysate (Meripro 500) was supplied by Tereos Syral, corn protein isolate was supplied by Cargill, and pea protein concentrate (Nutralys S85F) was supplied by Kellogg. Participants were allocated to a treatment according to a block randomization list performed using a computerized randomizer (http://www.randomization.com/). An independent researcher was responsible for random assignment (*n* = 12 per group) and preparation of the study treatment beverages, which were sequentially numbered according to subject number. The beverages were prepared in nontransparent protein shakers.

### Diet and physical activity

Participants refrained from sports and strenuous physical activities (e.g., lifting heavy weights), and alcohol consumption for 3 d prior to the experimental trial. In addition, all participants were instructed to complete a food and activity record for 3 d prior to the experimental trial. (See **Supplemental Table 1** for an overview of participants’ habitual food intake in the 3 d prior to the experimental trial.) The evening before the trial, all participants consumed a standardized dinner containing 2.8 MJ, with 65% energy provided as carbohydrate, 20% as fat, and 15% as protein, before 22:00, after which they remained fasted.

### Experimental protocol

The procedures applied in this trial have previously been described elsewhere (26). At ∼07:30, participants arrived at the laboratory in an overnight postabsorptive state. A cannula was inserted into an antecubital vein for stable isotope amino acid infusion. A second cannula was inserted retrogradely into a dorsal hand vein on the contralateral arm for arterialized blood sampling. To obtain arterialized blood samples, the hand was placed in a hot box (60°C) for 10 min prior to blood sample collection.

After taking a baseline blood sample (*t* = −180 min), the plasma phenylalanine pool was primed with a single dose of l-[ring-^13^C_6_]-phenylalanine (2.25 μmol/kg). Thereafter, a continuous intravenous infusion of l-[ring-^13^C_6_]-phenylalanine (0.05 μmol/kg/min) was initiated (*t* = −180 min) using a calibrated IVAC 598 pump. Subsequently, arterialized blood samples were collected at *t* = −90, −60, and −30 min relative to beverage ingestion. At *t* = 0 min an arterialized blood sample was obtained and a biopsy was collected from the vastus lateralis muscle. Immediately following the muscle biopsy, participants ingested a 400-mL beverage corresponding to their randomized treatment allocation, that is, MILK (*n* = 12) or PLANT-BLEND (*n* = 12). To minimize dilution of the steady-state plasma l-[ring-^13^C_6_]-phenylalanine precursor pool, the phenylalanine content of the protein drink was enriched with 3.85% l-[ring-^13^C_6_]-phenylalanine. Frequent arterialized blood samples were then collected for 300 min after protein ingestion. A second and third biopsy from the vastus lateralis muscle were collected at *t* = 120 and *t* = 300 min to determine postprandial skeletal muscle protein synthesis rates over the 0–120, 120–300, and 0–300 min postprandial periods. Muscle biopsies were obtained with the use of a 5 mm Bergström needle (27), custom-adapted for manual suction, and blood samples were collected into EDTA-containing tubes, according to the procedures described previously (26). For a schematic representation of the infusion protocol, see **Supplemental Figure 2**.

### Protein powder analysis

Batch-specific nitrogen contents for milk protein concentrate, wheat protein hydrolysate, corn protein isolate, and pea protein concentrate were provided by the manufacturer. The protein content of the milk protein was determined as nitrogen content × 6.38, the protein content of wheat protein powder was determined as nitrogen content × 5.7 (28, 29), and the protein content of corn and pea protein were determined as nitrogen × 6.25. Amino acid contents of the protein powders were determined by acid hydrolysis in triplicate, and subsequent analysis of the free amino acids using ultra-performance liquid chromatography-mass spectrometry (UPLC-MS; ACQUITY UPLC H-Class with QDa; Waters), as previously described (26). The amino acid composition of the protein powders and the protein blend are presented in **Table 2**.

**TABLE 2:** Amino acid composition of protein or protein blend consumed^1^

	MILK	PLANT-BLEND^2^
Alanine	0.9	1.2
Arginine	0.8	1.0
Aspartic acid	1.8	1.4
Cystine	0.1	0.2
Glutamic acid	5.1	7.6
Glycine	0.5	1.0
Histidine	0.6	0.5
Isoleucine	0.9	0.6
Leucine	2.4	2.4
Lysine	2.0	0.7
Methionine	0.7	0.4
Phenylalanine	1.2	1.4
Proline	2.9	3.0
Serine	1.2	1.4
Threonine	0.9	0.7
Tyrosine	0.6	0.5
Valine	1.1	0.7
TAA	23.8	24.7
EAA	9.8	7.4
BCAA	4.4	3.7
Nitrogen content, %	13.4	13.9
Protein content, %	85.5^3^	83.2^4^

1 Values for amino acid contents are in grams per 30 g protein. MILK: 30 g milk protein; PLANT-BLEND: 15 g wheat protein + 7.5 g corn protein + 7.5 g pea protein. BCAA, branched chain amino acids; EAA, essential amino acids; TAA, total amino acids.

2 Values are obtained by averaging the measured values for wheat, corn, and pea protein in a 2:1:1 ratio.

3 Nitrogen-to-protein conversion factor: 6.38.

4 Nitrogen-to-protein conversion factor: 5.7 for wheat and 6.25 for corn and pea protein.

### Plasma analysis

Plasma glucose and insulin concentrations were analyzed using commercially available kits (ref. no. A11A01667, Glucose HK CP, ABX Diagnostics; and ref. no. HI-14 K, Millipore, respectively). Plasma amino acid concentrations were determined by UPLC-MS, as previously described (26). Plasma l-[ring-^13^C_6_]-phenylalanine enrichments were determined by GC-MS (Agilent 7890A GC/5975C MSD; Agilent Technologies), as previously described (26).

Basal muscle protein synthesis rates were assessed to confirm that protein ingestion increases muscle protein synthesis rates. The single biopsy approach was applied to assess postabsorptive muscle protein synthesis rates without the need to collect an additional muscle biopsy, as previously described (26, 30).

### Muscle analysis

Muscle analysis for the determination of muscle protein–bound l-[ring-^13^C_6_]-phenylalanine enrichments by gas chromatography-combustion-isotope ratio mass spectrometry (GC-IRMS) has previously been explained in detail (26). In short, a piece of wet muscle (∼50–70 mg) was homogenized and prepared and a myofibrillar protein–enriched fraction was obtained by removal of the collagen-enriched fraction. Subsequently, the amino acids were liberated from the myofibrillar protein–enriched fraction by adding 2 mL 6M HCl and heating to 110°C for 16 h. The amino acids from the resulting dried myofibrillar protein–enriched fractions were liberated by adding 2 mL 6M HCl and heating to 110°C for 16 h, passing over a cation-exchange resin column (AG 50W-X8, mesh size: 100–200, ionic form: hydrogen; Bio-Rad Laboratories), and derivatized to their *N*(*O,S*)-ethoxycarbonyl ethyl esters. The ratio of ^13^C/^12^C of myofibrillar protein–bound phenylalanine was determined using GC-IRMS.

### Calculations

Net incremental area under curve (iAUC) was determined for plasma amino acid concentrations during the 5-h postprandial period following protein ingestion. The iAUC was calculated using the trapezoid rule, with plasma concentrations before beverage ingestion (*t* = 0 min) serving as baseline.

Myofibrillar protein fractional synthetic rates (FSRs, percentage per hour) were calculated by the standard precursor-product equation (31), as previously described (26).

### Outcome measures

Myofibrillar FSR over the entire (i.e., 0–300 min) postprandial period, comparing MILK with PLANT-BLEND, was defined as the primary outcome measure. Secondary outcome measures included myofibrillar FSR in the early (i.e., 0–120 min) and late (i.e., 120–300 min) postprandial periods, plasma glucose, insulin, and amino acid concentrations over time, and plasma amino acid iAUC. Plasma glucose, insulin, and amino acid peak concentrations and time to peak were tertiary outcomes.

### Statistical analysis

A power calculation was performed with differences in postprandial myofibrillar FSRs between the 2 treatments as primary outcome measure. Based on previous work in this area, a sample size of 12 participants per treatment, including a 10% dropout rate, was calculated using a power of 80%, a significance level of 0.05, a difference in FSR of 0.008%/h (or ∼20% when expressed as relative difference; e.g., 0.040 compared with 0.048%/h) (25), and a within-group SD of 0.0065%/h (or ∼16%) (32, 33). Participants’ characteristics were analyzed by 2-samples *t* test. Plasma glucose, insulin, and amino acid concentrations and amino acid enrichments over time were compared between groups using a 2-factor (*P*-interaction) repeated measures ANOVA, with time as within-subjects factor, and treatment as between-subjects factor. In case a significant time × treatment interaction was observed, post hoc analyses were performed to determine significant differences between treatments for each time point. Within treatments, repeated measures analyses were performed to evaluate which time points were increased above baseline (before protein intake). Plasma glucose, insulin, and amino acid concentrations, expressed as peak values, time to peak, and iAUC, were analyzed by 2-samples *t* test to locate differences between groups. Basal postabsorptive (−180 to 0 min), and postprandial myofibrillar protein synthesis rates during the early (0–120 min), late (120–300 min), and entire (0–300 min) postprandial periods were analyzed by 2-samples *t* test. Statistical analyses were performed with a software package (IBM SPSS statistics for Windows, version 26.0; IBM Corp). Means were considered to be significantly different for *P* values < 0.05. Data are expressed as means ± SD; additionally, for the main outcome parameter (postprandial muscle protein FSR), and aggregate EAA, leucine, lysine, and methionine iAUC, the estimated differences ± SD with 95% CIs are provided. Except for plasma insulin concentrations (*n* = 11 for MILK), no missing values were present for any of the outcome parameters.

## Results

### Participants’ characteristics

Twenty-four healthy, recreationally active males (24 ± 4 y; 1.78 ± 0.07 m; 71.2 ± 8.7 kg) volunteered to participate in this parallel-group, double-blind, randomized controlled trial (**Table 1**).

### Plasma glucose and insulin concentrations

**FIGURE 1:**
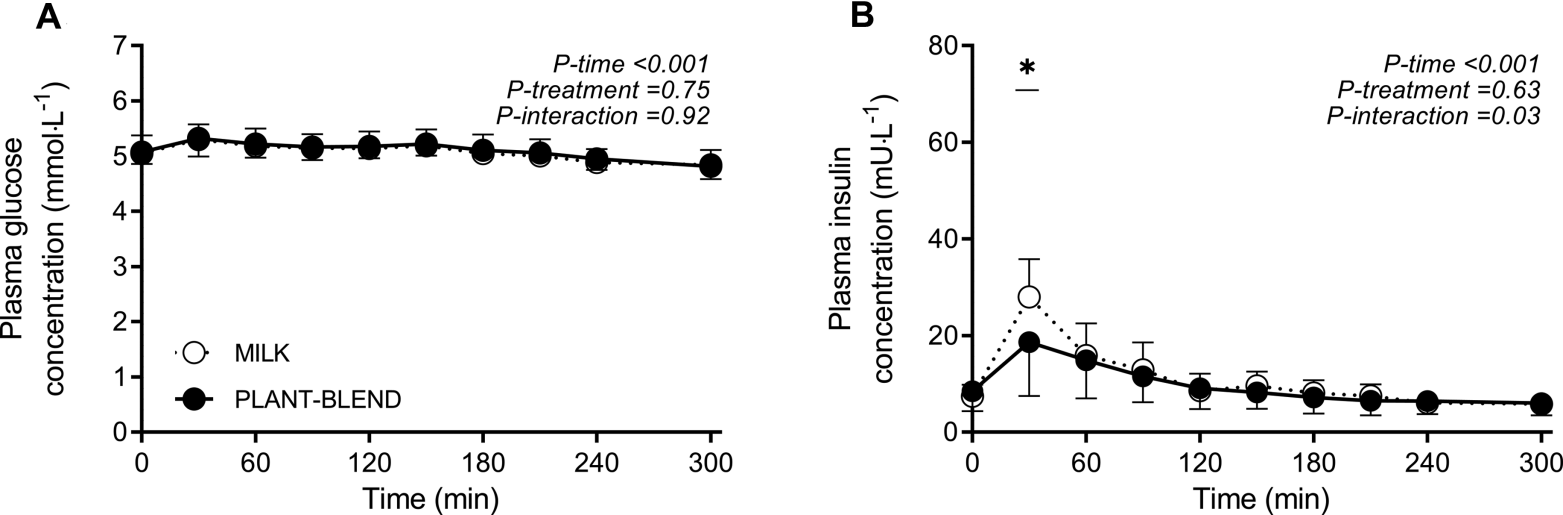
Postprandial plasma glucose (A) and insulin (B) concentrations during the 5-h period following the ingestion of MILK vs. PLANT-BLEND in healthy young males (*n* = 12 per group). Time 0 min represents time of beverage intake. MILK: 30 g milk protein; PLANT-BLEND: 15 g wheat + 7.5 g corn + 7.5 g pea protein. Values represent means ± SD; 2-factor repeated measures ANOVA with time as within-subjects variable and interventional drink (treatment) as between-subjects variable.

No significant changes or differences between treatments in plasma glucose concentrations were observed following protein ingestion (*P*-interaction = 0.92; **Figure 1**A). Plasma insulin concentrations increased following protein ingestion, with a greater initial rise following MILK compared with PLANT-BLEND ingestion (*P*-interaction = 0.03; 1 missing value MILK, *n* = 11; Figure 1B). However, peak plasma insulin concentrations did not differ between treatments (28 ± 8 compared with 19 ± 11 mU/L, respectively; 2-sample *t* test: *P* = 0.15). Postprandial plasma insulin availability (iAUC) was greater following MILK compared with PLANT-BLEND ingestion (1.06 ± 0.33 compared with 0.50 ± 57 mU·300 min·L^−1^, respectively; 2-sample *t* test: *P* < 0.05).

### Plasma amino acid concentrations

**FIGURE 2:**
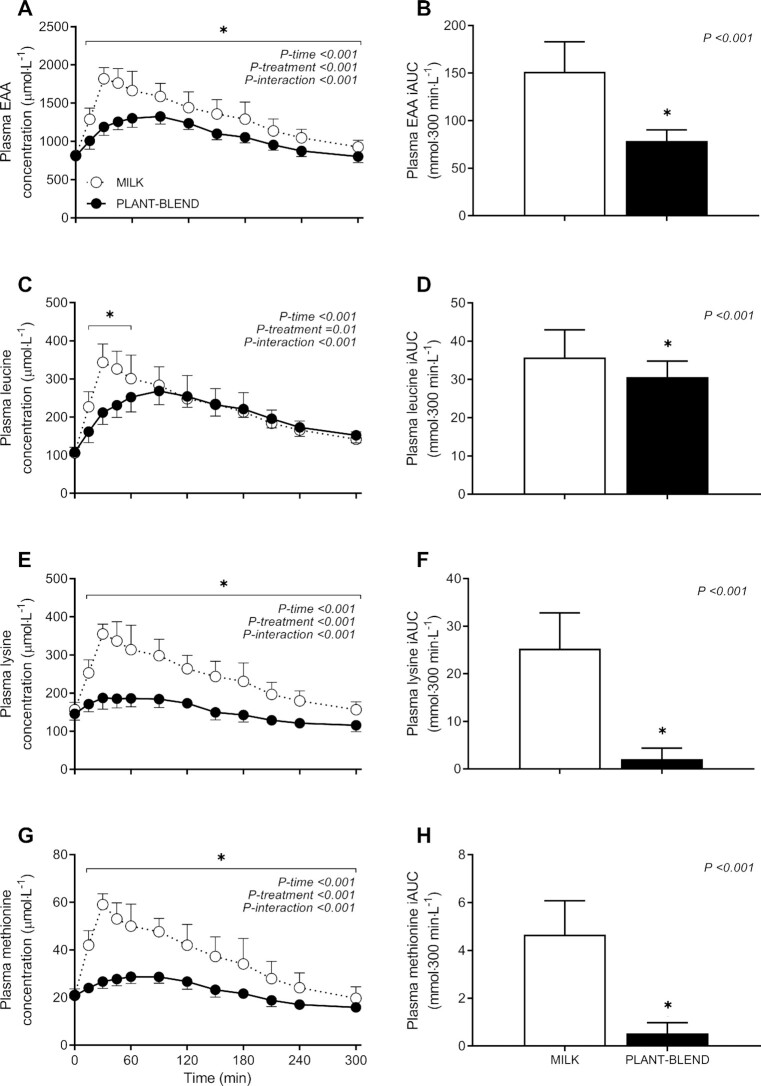
Postprandial plasma EAA (A), leucine (C), lysine (E), and methionine (G) concentrations during the 5-h postprandial period following the ingestion of MILK vs. PLANT-BLEND in healthy young males (*n* = 12 per group). Time 0 min represents time of beverage intake. Panels B, D, F, and H represent the 0–5-h iAUC following protein ingestion. MILK: 30 g milk protein; PLANT-BLEND: 15 g wheat + 7.5 g corn + 7.5 g pea protein. Values represent means ± SD; *denotes significantly different between intervention groups (*P* < 0.05). Two-factor repeated measures ANOVA with time as within-subjects variable and interventional drink (treatment) as between-subjects variable. EAA, essential amino acid; iAUC, incremental area under curve.

Plasma EAA concentrations increased following protein ingestion, with a more rapid and greater rise in circulating EAA concentrations following MILK compared with PLANT-BLEND ingestion (*P*-interaction < 0.001; **Figure 2**A). Plasma EAA concentrations increased for, respectively, 300 and 240 min after MILK and PLANT-BLEND ingestion. In line with the significant time × treatment interaction, peak plasma EAA concentrations were reached at an earlier point in time following MILK compared with PLANT-BLEND ingestion (at 36 ± 10 and 75 ± 26 min after protein ingestion, respectively; 2-sample *t* test: *P* < 0.001), reaching concentrations of 1870 ± 124 and 1370 ± 93 μmol/L, respectively (2-sample *t* test: *P* < 0.001). The overall increase in plasma EAA availability over the entire 300-min postprandial period, expressed as iAUC, was ∼2-fold greater for MILK compared with PLANT-BLEND [151 ± 31 compared with 79 ± 12 mmol·300 min·L^−1^; 2-sample *t* test: *P* < 0.001; estimated mean difference = 72.6 ± 33.5 (95% CI: 52.6, 92.7) mmol·300 min·L^−1^; Figure 2B].

The postprandial increase in plasma leucine concentrations following protein ingestion (Figure 2C) differed between MILK compared with PLANT-BLEND (*P*-interaction < 0.001). Plasma leucine concentrations increased for the entire 300-min postprandial period following ingestion of both protein drinks. In line with the significant time × treatment interaction, peak plasma leucine concentrations were ∼25% greater for MILK compared with PLANT-BLEND (353 ± 45 compared with 283 ± 22 μmol/L, respectively; 2-sample *t* test: *P* < 0.001) and were reached ∼1 h earlier (46 ± 43 and 113 ± 46 min after protein ingestion, respectively; 2-sample *t* test: *P* = 0.001). The overall increase in plasma leucine availability over the entire 300-min postprandial period, expressed as iAUC, was ∼16% greater for MILK compared with PLANT-BLEND [36 ± 7 compared with 31 ± 4 mmol·300 min·L^−1^; 2-sample *t* test: *P* = 0.046; estimated mean difference: 5.1 ± 8.3 (95% CI: 0.1, 10.1) mmol·300 min·L^−1^; Figure 2D].

The postprandial increase in plasma lysine concentrations following protein ingestion was significantly greater following MILK compared with PLANT-BLEND ingestion (*P*-interaction < 0.001; Figure 2E). Plasma lysine concentrations increased for 240 and 120 min after MILK and PLANT-BLEND consumption, respectively. In line with the significant time × treatment interaction, peak plasma lysine concentrations were 85% greater following MILK compared with PLANT-BLEND ingestion (370 ± 29 compared with 201 ± 24 μmol/L, respectively; 2-sample *t* test: *P* < 0.001), and were reached earlier (34 ± 7 compared with 60 ± 34 min after protein ingestion; 2-sample *t* test: *P* = 0.02). Peak plasma lysine concentrations increased ∼137% above baseline values for MILK, but only increased ∼38% above baseline for PLANT-BLEND. Consequently, the overall increase in plasma lysine availability over the entire 300-min postprandial period, expressed as iAUC, was much greater for MILK compared with PLANT-BLEND [25 ± 8 compared with 2 ± 2 mmol·300 min·L^−1^; 2-sample *t* test: *P* < 0.001; estimated mean difference: 23.2 ± 7.9 (95% CI: 18.5, 28.0) mmol·300 min·L^−1^; Figure 2F].

The postprandial increase in plasma methionine concentration following protein ingestion was significantly greater following MILK compared with PLANT-BLEND ingestion (*P*-interaction < 0.001; Figure 2G). Plasma methionine concentrations increased for 240 and 150 min after MILK and PLANT-BLEND consumption, respectively. In line with the significant time × treatment interaction, peak plasma methionine concentrations were ∼104% greater for MILK compared with PLANT-BLEND (60 ± 5 and 30 ± 2 μmol/L; 2-sample *t* test: *P* < 0.001) and were reached earlier (34 ± 9 compared with 69 ± 24 min after protein ingestion; 2-sample *t* test: *P* < 0.001). As a result, peak plasma methionine concentrations increased ∼190% above baseline values for MILK, but only increased ∼42% above baseline values for PLANT-BLEND. The overall increase in plasma methionine availability over the entire 5-h postprandial period, expressed as iAUC, was severalfold greater for MILK compared with PLANT-BLEND [4.7 ± 1.4 compared with 0.5 ± 0.4 mmol·300 min·L^−1^; 2-sample *t* test: *P* < 0.001; estimated mean difference: 4.1 ± 1.5 (95% CI: 3.3, 5.0) mmol·300 min·L^−1^; Figure 2H].

In general, postprandial increases in plasma amino acid concentrations revealed significant differences over time following MILK compared with PLANT-BLEND ingestion for most amino acids (**Supplemental Figure 3**; *P*-interaction < 0.05). The postprandial increases in plasma isoleucine, threonine, tryptophan, tyrosine, and valine availability over the entire 5-h postprandial period (iAUC) were greater for MILK compared with PLANT-BLEND (2-sample *t* test: *P* < 0.05), whereas only for glycine was plasma availability lower for MILK compared with PLANT-BLEND (2-sample *t* test: *P* < 0.01; Supplemental Figure 3).

### Plasma free and muscle protein–bound l-[ring-^13^C_6_]-phenylalanine enrichments

**FIGURE 3:**
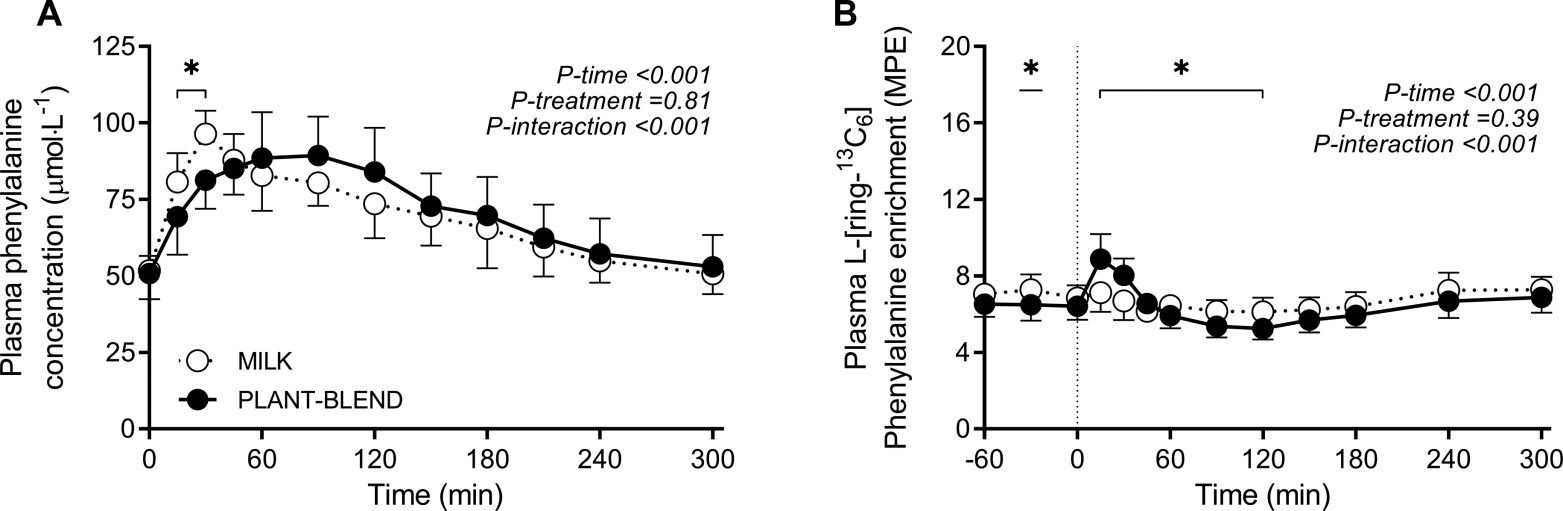
Postprandial plasma phenylalanine concentrations (A) and plasma l-[ring-^13^C_6_]-phenylalanine enrichments (B) during the 5-h postprandial period following the ingestion of MILK vs. PLANT-BLEND in healthy young males (*n* = 12 per group). Time 0 min represents time of beverage intake. MILK: 30 g milk protein; PLANT-BLEND: 15 g wheat + 7.5 g corn + 7.5 g pea protein. Values represent means ± SD; *denotes significantly different between intervention groups (*P* < 0.05). Two-factor repeated measures ANOVA with time as within-subjects variable and interventional drink (treatment) as between-subjects variable. MPE, mole percentage excess.

Plasma phenylalanine concentrations and l-[ring-^13^C_6_]-phenylalanine enrichments over time are presented in **Figure 3**A and B, respectively. Plasma l-[ring-^13^C_6_]-phenylalanine enrichments were lower following MILK compared with PLANT-BLEND ingestion during the early postprandial period (*P-*interaction < 0.001). Weighted mean plasma l-[ring-^13^C_6_]-phenylalanine enrichments averaged 7.11 ± 0.65 and 6.48 ± 0.70 mole percentage excess (MPE) during the basal postabsorptive period (2-sample *t* test: *P* = 0.04), and 6.64 ± 0.53 and 6.32 ± 0.55 MPE throughout the 5-h postprandial period (2-sample *t* test: *P* = 0.16) following MILK and PLANT-BLEND ingestion, respectively.

Myofibrillar protein–bound l-[ring-^13^C_6_]-phenylalanine enrichments were higher following ingestion of MILK and PLANT-BLEND, from 0.0032 ± 0.0031 and 0.0045 ± 0.0045 MPE at *t* = 0 min, to 0.0115 ± 0.0041 and 0.0145 ± 0.0076 MPE at *t* = 120 min, reaching 0.0214 ± 0.0049 and 0.0250 ± 0.0083 MPE at *t* = 300 min after protein ingestion, respectively. The plasma free and muscle protein–bound l-[ring-^13^C_6_]-phenylalanine enrichments were subsequently used to calculate muscle protein synthesis rates.

### Muscle protein synthesis rates

**FIGURE 4:**
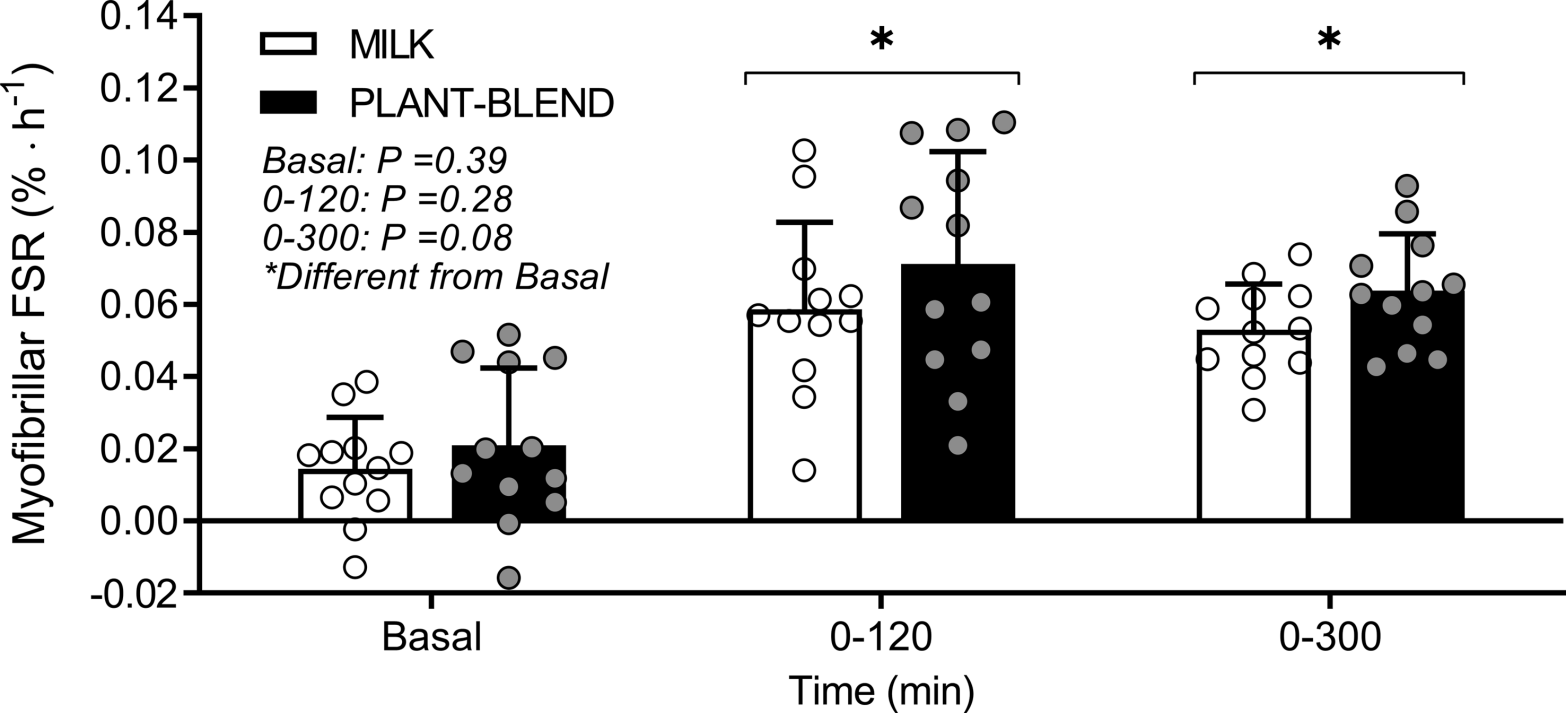
Myofibrillar protein fractional synthetic rates (FSR) at different time periods prior to and following ingestion of MILK vs. PLANT-BLEND in healthy young males (*n* = 12 per group). MILK: 30 g milk protein; PLANT-BLEND: 15 g wheat + 7.5 g corn + 7.5 g pea protein. Values represent means ± SD. *Denotes significantly different from basal; 2-samples *t* test: *P* < 0.05. Two-samples *t* test: *P* = 0.39, *P* = 0.28, and *P* = 0.08 for basal, 0–120 min, and 0–300 min, respectively. No differences were observed between treatments.

Postabsorptive myofibrillar protein FSRs averaged 0.014 ± 0.014 and 0.021 ± 0.021%/h in the MILK and PLANT-BLEND group, with no differences between treatments (2-sample *t* test: *P* = 0.39). Protein ingestion increased myofibrillar protein synthesis rates to 0.059 ± 0.024 and 0.071 ± 0.031%/h during the early postprandial period (0–120 min) and to 0.049 ± 0.017 and 0.058 ± 0.015%/h during the late postprandial period (120–300 min) in MILK and PLANT-BLEND, respectively (2-sample *t* test: *P* < 0.05). Postprandial muscle protein synthesis rates averaged 0.053 ± 0.013 and 0.064 ± 0.016%/h assessed over the entire 5-h postprandial period (**Figure 4**). Postprandial myofibrillar protein synthesis rates did not differ between MILK compared with PLANT-BLEND for the early (0–120 min; 2-sample *t* test: *P* = 0.58), late (120–300 min; 2-sample *t* test: *P* = 0.20), and entire (0–300 min; 2-sample *t*-test: *P* = 0.08) postprandial period. The estimated differences for the muscle protein synthesis rates were respectively: −0.0125 ± 0.0393%/h (95% CI: −0.03609, 0.0110%/h) for the early; −0.0086 ± 0.0223%/h (95% CI: −0.0220, 0.0048%/h) for the late; and −0.0107 ± 0.0202%/h (95% CI: −0.2284, 0.0014%/h) for the entire postprandial period.

## Discussion

The present study shows that ingestion of a wheat, corn, and pea protein blend strongly increases muscle protein synthesis rates in healthy young males. The muscle protein synthetic response to the ingestion of 30 g of this plant-derived protein blend did not differ from the ingestion of an equivalent amount of milk protein, despite an attenuated postprandial rise in circulating plasma EAA concentrations.

Plant-derived proteins are known to have specific amino acid deficiencies according to the WHO/FAO/UNU requirements (24), and are generally low in EAA content and leucine in particular (19). Combining different plant-derived proteins allows us to compose a protein blend with a more balanced amino acid profile, with no apparent amino acid deficiencies. We combined wheat-, corn-, and pea-derived protein in a 2:1:1 ratio to provide a plant-derived protein blend with an amino acid profile that resembles high-quality animal-derived proteins, such as milk protein (Table 2). With leucine being one of the key amino acids driving the anabolic response to protein ingestion (4–8), we included an ample amount of corn protein to compose a plant-derived protein blend with a leucine content (8%) well above the WHO/FAO/UNU leucine requirements (5.9%) (24). Although we were not able to provide EAA (27%), lysine (4.5%), and methionine (1.6%) contents fully compliant with the WHO/FAO/UNU requirements (29), the protein blend did provide an EAA content of no less than 25%, a lysine content ∼2-fold higher than wheat and corn protein on their own, and a methionine content that was ∼3-fold higher than pea protein on its own. This demonstrates that blending different plant-derived proteins can effectively improve the amino acid composition far beyond the composition of their individual proteins.

In the present study, the leucine content of the plant-derived protein blend was matched with milk protein (2.4 g), but the EAA (7.4 compared with 9.8 g), lysine (0.7 compared with 2.0 g), and methionine (0.4 compared with 0.7 g) contents remained below the levels observed in the milk protein (Table 2). These differences in amino acid composition translated into lower peak plasma EAA, lysine, and methionine concentrations and a lower postprandial plasma amino acid availability (Figure 2). Despite the matching leucine contents, peak plasma leucine concentrations and iAUC were lower following ingestion of the blend when compared with the milk protein (Figure 2). The observed differences in postprandial plasma amino acid profiles tend to agree with previous work showing an attenuated postprandial rise in circulating plasma amino acids following ingestion of plant-derived protein isolates and concentrates when compared with the ingestion of an equivalent amount of animal-based protein (26, 34). Though we can only speculate on the mechanisms responsible, there are ample reports suggesting that differences in protein structure and function and the presence of antinutritional factors can compromise protein digestion and amino acid absorption, and/or modulate splanchnic extraction of protein-derived amino acids (35–37).

To assess the impact of these differences in postprandial plasma amino acid responses on the postprandial stimulation of muscle protein synthesis, we combined a primed continuous l-[ring-^13^C_6_]-phenylalanine infusion with the collection of muscle biopsies. The postprandial rise in circulating plasma EAAs following ingestion of the plant-derived protein blend strongly increased muscle protein synthesis rates when compared with basal, postabsorptive values (Figure 4). The response tended to be of a similar magnitude when compared with previous responses observed following ingestion of similar amounts of high-quality animal-derived proteins (25, 38, 39). In the present study, we included a control treatment in which 30 g high-quality milk protein concentrate was ingested. Interestingly, despite the lower postprandial plasma amino acid availability following ingestion of the plant-derived protein blend when compared with the milk protein ingestion, we observed no differences in postprandial muscle protein synthesis rates. In fact, there was a trend for postprandial muscle protein synthesis rates to increase to a greater extent following the ingestion of the plant-derived protein blend when compared with milk protein (*P* = 0.08; Figure 4). The present study extends on previous studies comparing the anabolic properties of dairy plus plant–based protein blends with dairy protein (21, 23, 26), by showing that even an exclusively plant-derived protein blend can be as effective as a high-quality animal protein in stimulating muscle protein synthesis in vivo in healthy young adults.

There has been growing interest in consumption of a more plant-based diet and the application of plant-derived proteins in our food as a means to replace animal-based food products. However, individual plant-derived foods are regarded as a lesser quality protein source because of their lower digestibility, low EAA content, and/or specific amino acid deficiencies (17, 19, 22). However, these deficiencies can be overcome by composing blends of complementary plant-based protein sources or plant-derived protein isolates and concentrates, making the overall protein quality comparable to a high-quality animal-based protein source (22). Therefore, plant-derived protein blends can be effectively applied in the development of high-quality plant-based products, or in composing high-quality plant-based protein meals. Here, we show that ingestion of a plant-derived protein blend does not compromise the postprandial muscle protein synthetic response when compared with the consumption of an equivalent amount of a high-quality animal-derived reference protein (Figure 4). We provided our participants with 30 g protein, containing no less than 2.4 g leucine in both protein groups. Consequently, we provided an amount of leucine similar to that shown to maximally stimulate resting postprandial muscle protein synthesis rates in young adults when provided with 20 g whey protein (2.2 g) (25). This allowed us to evaluate the true anabolic potential of plant-derived protein sources, which are usually low in leucine. Thereby, 30 g protein is still a feasible amount of protein to ingest in a meal, whereas ingestion of much higher dosages of protein can become challenging. Therefore, if differences in the muscle protein synthetic response would already have been apparent with a protein intake of 30 g, while providing a sufficient amount of leucine, the feasibility of this protein blend for stimulating muscle protein synthesis would have been questionable. Consequently, given the amount of protein and leucine provided, we might have maximally stimulated the muscle protein synthetic response for both intervention groups. In line, the lower plasma aminoacidemia following ingestion of the plant blend compared with milk protein, could already have been sufficient to maximally stimulate muscle protein synthesis. We can only speculate whether differences in the muscle protein synthetic response to the consumption of plant-derived proteins (26, 34) and plant-derived protein blends when compared with animal-derived protein become apparent when (much) lower amounts of protein are ingested. Providing less protein could result in lower postprandial plasma amino acid availability and, as such, could lead to the detection of differences between plant- and animal-derived proteins in their capacity to stimulate muscle protein synthesis. The latter can be attributed to specific amino acid deficiencies in the plant-derived proteins. However, the use of protein blends compensates for specific amino acid deficiencies, so we speculate that lower amounts would still show no differences in anabolic properties of this protein blend when compared with milk protein. This rationale seems to be supported by the fact that we even observed a trend for higher postprandial protein synthesis rates following ingestion of the plant-derived protein blend compared with the animal-derived protein source. More work will be needed to assess the anabolic responses to the ingestion of plant-derived protein blends in older and more clinically compromised populations who typically consume less protein per serving and/or have a lower anabolic response to protein ingestion (40). Furthermore, it should be highlighted that this work focuses specifically on protein concentrates/isolates in order to provide insight in the potential of the proteins themselves. However, these findings might not directly translate to plant-based whole foods and food products. These products contain many other nutrients and antinutritional factors that can strongly impact protein digestion and amino acid absorption kinetics and, as such, are likely to restrict the postprandial anabolic response. Furthermore, protein extraction and the associated processing of plant-derived proteins can also affect bioavailability as well as biofunctionality of these proteins. Therefore, more research is needed to translate the current findings in a meal-based setting. Lastly, our data were obtained from healthy men and because there might (41, 42) or might not (43, 44) be differences in postprandial protein handling between men and women, the present data do not necessarily apply to females. Future research should include both male and female participants.

We conclude that ingestion of 30 g of a wheat- (50%), corn- (25%), and pea- (25%) derived protein blend increases muscle protein synthesis rates in healthy young males. The muscle protein synthetic response to the ingestion of a well-composed plant-derived protein blend can be as robust as an equivalent amount of a high-quality animal-derived protein. Balanced plant-derived protein blends can have anabolic properties that do not differ from high-quality animal-derived proteins.

## Supplementary Material

nxac222_Supplemental_File

## Data Availability

Data is available on request.

## Notes

The project is organized by and executed under the auspices of TiFN (Wageningen, The Netherlands), a public-private partnership on precompetitive research in food and nutrition. Funding for this research was obtained from Tereos (Marckolsheim, France), Cargill (Minneapolis, MN, USA), Kellogg (Battle Creek, MI, USA), and the Top-sector Agri&Food.

Author disclosures: PJMP, IWKK, JMS, LCPGMdG, LBV, TS and LJCvL have the following interests: This study was funded by TiFN, Wageningen, The Netherlands. The sponsors Tereos Syral (Marckolsheim, France), Cargill (Minneapolis, MN, USA), and Kellogg (Battle Creek, MI, USA) partly financed the project. The researchers are responsible for the study design, data collection and analysis, decision to publish, and preparation of the manuscript. The industrial partners have contributed to the project through regular discussion, and were involved in the study design. More specifically: the choice of interventional products which were produced by these sponsors. The funders had no role in data collection and analysis, decision to publish, or preparation of the manuscript. In addition: LJCvL and LBV have received research grants, consulting fees, speaking honoraria, or a combination of these from Friesland Campina, Tereos Syral, and Pepsico. The other authors report no conflicts of interest.

The researchers are responsible for the study design, data collection and analysis, decision to publish, and preparation of the manuscript. The industrial partners have contributed to the project through regular discussion, and were involved in the study design, and more specifically in the choice of interventional products which were produced by these partners. The funders had no role in data collection and analysis, decision to publish, or preparation of the manuscript.

Supplemental Table 1 and Supplemental Figures 1–3 are available from the “Supplementary data” link in the online posting of the article and from the same link in the online table of contents at https://academic.oup.com/jn/.

Abbreviations used: EAA, essential amino acid; FSR, fractional synthetic rate; GC-IRMS, gas chromatography-combustion-isotope ratio mass spectrometry; iAUC, net incremental area under curve; MILK, 30 g milk protein concentrate; MPE, mole percentage excess; PLANT-BLEND, 15 g wheat protein hydrolysate + 7.5 g corn protein isolate + 7.5 g pea protein concentrate; UNU, United Nations University; UPLC-MS, ultra-performance liquid chromatography-mass spectrometry.
