# Supplementary material for: The Muscle Protein Synthetic Response to the Ingestion of a Plant-Derived Protein Blend Does Not Differ from an Equivalent Amount of Milk Protein in Healthy Young Males
Source: J Nutr. 2022 Sep 28;152(12):2734–43. doi: 10.1093/jn/nxac222 (PMC9839989; doi:10.1093/jn/nxac222)
Supplement: nxac222_Supplemental_File [file nxac222_supplemental_file.docx]

| 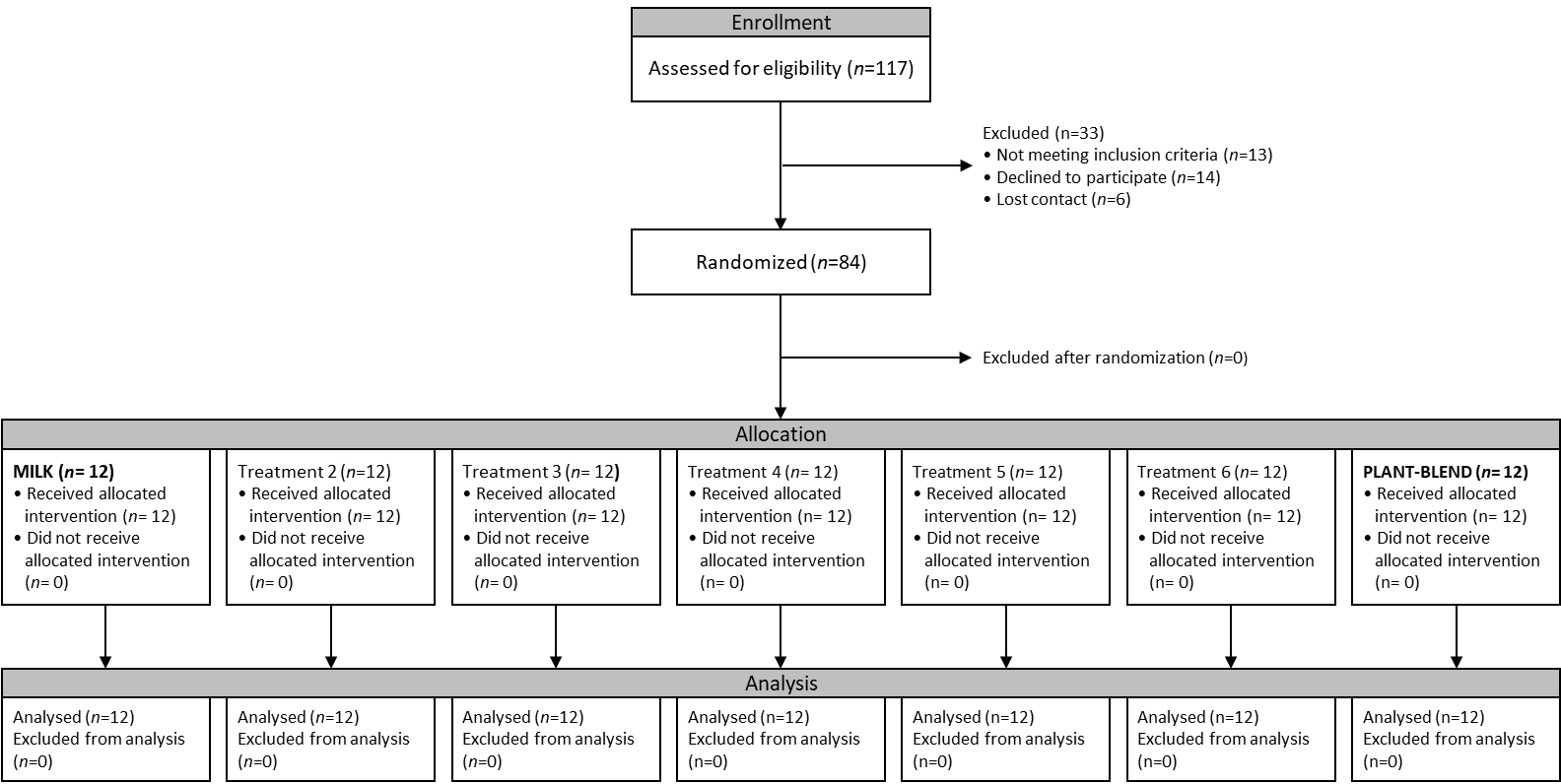 |
| --- |
| **Supplemental figure 1:** CONSORT flow diagram. CONSORT, Consolidated Standards of Reporting Trials. The current study was part of a larger trial with a total of 7 parallel groups (*n* = 12 per group) as indicated in the flow diagram. MILK (30 g milk protein), PLANT-BLEND (15 g wheat protein + 7.5 g corn protein + 7.5 g pea protein). |
| 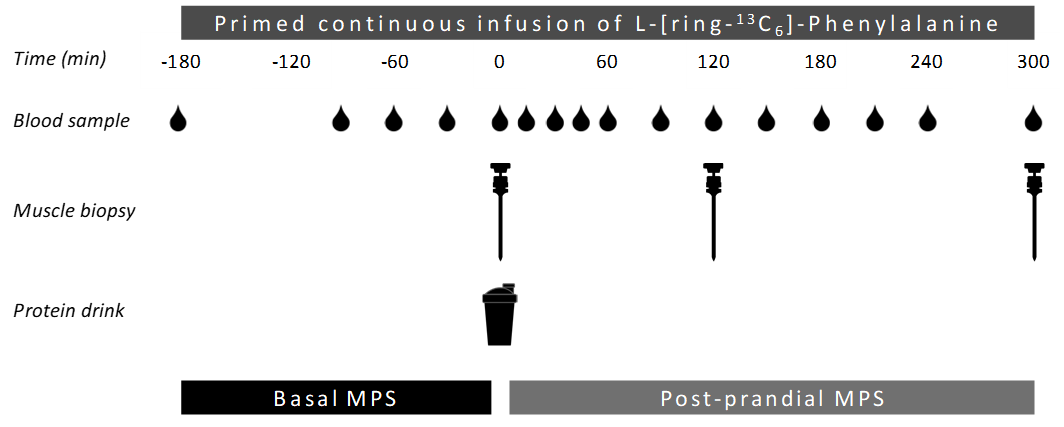 |
| **Supplemental figure 2:** Schematic representation of the experimental design. |

|  |
| --- |
| **Supplemental figure 3:** Figure to be continued on next page |
|  |
| **Supplemental figure 3:** Figure to be continued on next page |
|  |
| **Supplemental figure 3:** Figure to be continued on next page |
|  |
| **Supplemental figure 3:** Figure to be continued on next page |
|  |
| **Supplemental figure 3:** Post-prandial plasma amino concentrations during the 300 min post-prandial period following the ingestion of MILK vs PLANT-BLEND (*n*=12 per group). Time 0 min represents time of beverage intake. Panels B, D, F, H, J, L, N, P, R, T, V, X, Z, AB, AD, AF, AH, AJ, AL represent the 0-5 h incremental area under curve (iAUC) following protein ingestion. MILK (30 g milk protein), PLANT-BLEND (15 g wheat protein + 7.5 g corn protein + 7.5 g pea protein). Values represent means ± standard deviation; *significantly different between interventions (*P*<0.05). Repeated measures ANOVA with time as within-subject variable and interventional drink (treatment) as between-subject variable, and two samples *t*-test were used to determine differences between groups. |

| **Supplemental Table 1:** Average 3 day dietary intake of study participants | | | | | |
| --- | --- | --- | --- | --- | --- |
|  | MILK | |  | PLANT-BLEND | |
|  | *Mean* | *SD* |  | *Mean* | *SD* |
| Energy (MJ∙d^-1^) | 9.3 | 2.2 |  | 9.6 | 2.2 |
| Carbohydrate (g∙d^-1^) | 267 | 63 |  | 274 | 56 |
| Fat (g∙d^-1^) | 78 | 27 |  | 88 | 27 |
| Protein (g∙d^-1^) | 97 | 29 |  | 94 | 13 |
| Energy (kJ∙kg^-1^∙d^-1^) | 131 | 26 |  | 136 | 32 |
| Carbohydrate (g∙kg^-1^∙d^-1^) | 3.8 | 0.9 |  | 3.9 | 0.9 |
| Fat (g∙kg^-1^∙d^-1^) | 1.1 | 0.3 |  | 1.2 | 0.4 |
| Protein (g∙kg^-1^∙d^-1^) | 1.3 | 0.4 |  | 1.3 | 0.2 |
| Carbohydrate (% total energy) | 50 | 7 |  | 49 | 4 |
| Fat (% total energy) | 33 | 8 |  | 34 | 6 |
| Protein (% total energy) | 18 | 3 |  | 17 | 4 |
| Values represent mean ± standard deviation. *n*=12 per nutritional intervention group. MILK: 30 g milk protein, PLANT-BLEND: 15 g wheat protein + 7.5 g corn protein + 7.5 g pea protein. Independent samples *t*-test for MILK *vs* PLANT-BLEND. Independent samples *t-test* between groups: all *P*>0.05. 3 Day food records were analyzed using “Mijn Eetmeter” (https://mijn.voedingscentrum.nl /nl/eetmeter/), online software available from the Netherlands Nutrition Centre. | | | | | |
